# Co-administration of GnRH agonists with vaginal progesterone compared to vaginal progesterone in luteal phase support of the frozen-thawed embryo transfer cycle: An RCT

**DOI:** 10.18502/ijrm.v19i10.9817

**Published:** 2021-11-04

**Authors:** Afsoon Zareii, Sara Davoodi, Mahshid Alborzi, Marzieh Eslami Moghadam, Elham Askary

**Affiliations:** ^1^Infertility Division, Infertility Research Center, School of Medicine, Shiraz University of Medical Sciences, Shiraz, Iran.; ^2^Infertility Division, Infertility Research Center, Department of Obstetrics and Gynecology, School of Medicine, Shiraz University of Medical Sciences, Shiraz, Iran.; ^3^Infertility Division, School of Medicine, Jahrom University of Medical Sciences, Jahrom, Iran.; ^4^School of Medicine, Shiraz University of Medical Sciences, Shiraz, Iran.; ^5^Department of Obstetrics and Gynecology, Laparoscopy Research Center, School of Medicine, Shiraz University of Medical Sciences, Shiraz, Iran.

**Keywords:** Luteal phase, Fertilization in vitro, Embryo transfer.

## Abstract

**Background:**

Since progesterone alone does not seem to be enough for luteal phase support (LPS), especially in frozen embryo transfer (FET) cycles, so gonadotropin-releasing hormone agonist (GnRH-a) is suggested as an adjuvant therapy in combination with progesterone for LPS.

**Objective:**

To evaluate the effects of the administration of GnRH-a with vaginal progesterone compared to vaginal progesterone alone in luteal phase support of the frozen-thawed embryo transfer cycles.

**Materials and Methods:**

In this randomized controlled clinical trial, 240 infertile women who were candidates for FET were evaluated into two groups (n = 120/each). Group I received 400 mg vaginal progesterone twice a day from the time of transfer. The second group received vaginal progesterone and also 0.1 mg diphereline on days 0, 3, and 6 of FET for LPS. Finally, the clinical and ongoing pregnancy rates, and the implantation, and spontaneous abortion rates were compared in two groups.

**Results:**

Results showed that there was no significant difference between the mean age of women and the duration of infertility (p = 0.78, p = 0.58, respectively). There were no significant differences between groups in the terms of implantation and spontaneous abortion rates (p = 0.19, p = 0.31, respectively). However, in terms of clinical and ongoing pregnancy rates, the significant differences were seen between groups (p = 0.008 and p = 0.005, respectively).

**Conclusion:**

Co-administration of GnRH-a and vaginal progesterone in LPS may be superior to vaginal progesterone alone in women who underwent a frozen-selected embryo transfer cycle.

## 1. Introduction

The average prevalence of infertility is 1.9% in women aged 20-44 yr old (1). Despite the high cost of assisted reproductive technology, infertile couples have a high tendency to try these available procedures (2).

Nowadays, frozen embryo transfer (FET) in in vitro fertilization (IVF) cycles have become more popular as the technique is associated with lower rate of ovarian hyper stimulation syndrome and higher rate of endometrial-embryo synchrony, and may also associate with a greater IVF success rate (3-5). In addition, in contrast to fresh embryo transfer method, pregnancy complications such as preterm delivery, low birth weight, and small-for-gestational age will be also reduced (6, 7).

Given the important role of endometrial condition in the embryo implantation success rate and births and also the lack of enough progesterone for endometrial stability and receptivity in patients who are selected for HRT treatment for endometrial preparation, several studies have suggested some additional drugs to improve the success rate in FET and increase live births in these cycles by supporting the luteal phase such as exogenous progesterone, but this treatment does not seem to be enough (8-13).

The administration of gonadotropin-releasing hormone agonist (GnRH-a) for luteal phase support (LPS), especially in the middle of the luteal phase, can increase the implantation rate by preventing the premature decline of luteinizing hormone (LH) level and early regression of corpus luteum. It increases the number of LH receptors on the endometrial cells and the growth of endometrial pinopods (which lead to better endometrial thickness and receptivity FET cycles) (14-18). Also, administration of GnRH-a stimulates human chorionic gonadotropin (HCG) secretion from a human embryo which can activate the endocrine-paracrine pathways and increase the implantation rate in these cases (10-13, 15). The administration of a single dose of GnRH-a at the time of implantation for LPS is useful and in some studies has been investigated (19, 20). However, four recent systematic reviews have reported adding an extra dose of GnRH-a for better LPS in FET cycles (18, 21-23). The additional dose of GnRH-a was prescribed on the sixth day of the embryo transfer, two days before the expected day of implantation. This extra dose of GnRH-a administration simulates the natural cycle peak of progesterone during mid-part of the luteal phase by boosting endogenous LH and maintaining the proper level of progesterone for implantation (24-26).

The evaluation of the effect of GnRH-a administration on LPS is very limited in literature. Due to this reason and knowing the short duration effect of GnRH-a (24-36 hr) and the safety and effectiveness of the drug (15, 27), we designed a clinical trial study to compare the effect of GnRH-a + progesterone for LPS. The results of this study provide useful information on the effects of GnRH-a on the outcome of IVF in FET compare to vaginal progesterone alone.

## 2. Materials and Methods 

### Study design and participants

This randomized controlled clinical trial was conducted on 240 infertile women who were candidate for FET referred to the Department of Infertility in the Ghadir Mother & Child Hospital of Shiraz University of Medical Sciences (SUMS), Shiraz, Iran from September 2016 to March 2017.

The sample size was determined based on the following formula with 95% power and 0.05% = α error:


$$\frac{{\left(Z_{1-\frac{\alpha }{2}}\sqrt{2\bar{p}\bar{q}}+Z_{1-\beta }\sqrt{p_{1}q_{1}+p_{2}q_{2}}\right)}^{2}}{{\left(\delta \right)}^{2}}$$

240 participants were assigned randomly in to two groups; the random allocation method in this study was the permutation block randomization method, such that “A" represents the subject receiving the intervention (Drug), and “B" represents the subject who receives the placebo. This method is based on 60 blocks in 4 permutations (60 × 4 = 240), taking into account all possible quadruple permutations (AABB, ABAB, ABBA, BAAB, BBAA and BABA). Group I received 400 mg vaginal progesterone (Cyclogest) a day and group II received 0.1 mg diphereline on days 0, 3, and 6 of FET for LPS in addition to vaginal progestrone. The inclusion criteria were infertile women aged 20-40 yr with unexplained infertility, infertility due to fallopian tube factors, mild male factor, premature ovarian failure, or polycystic ovary syndrome, normal uterine cavity; and at least one-month gap since the previous IVF cycle. All women with endometriosis (stages III and IV), hydrosalpinx, infertility due to severe male factor, and recurrent implantation failure (at least three unsuccessful transfer cycles) were excluded.

### Intervention procedures

Women undergoing IVF with the agonist or antagonist protocol according to their condition were fertilized by IVF or ICSI after ovum pick-up. Approximately 16-19 hr after the fertilization, egg formation was confirmed by evaluating the presence of bipronuclear (2 PN) and was removed if microscopy was not seen. Then, the fertilized oocytes were classified by their quality so that on the third day of fertilization (66-68 hr), embryos with ≥ 8 cells with a fragmentation rate of < 20% were embedded as high quality (grade A) and embryos with lower quality were classified as group B, C, and D and were separated from each other. The embryos were frozen at the 8-cell stage (cleavage), using the slow freezing verification method, and the next protocol was maintained for each case in accordance with the standard protocol.

Endometrial preparation in women who did not have any intervention for at least one month before the transfer was started with 6 mg/day estradiol valerate (estradiol valerate tablet, 2 mg, Abooreyhan, Iran) from the second day of their menstrual cycle. The dosage of estradiol was increased until the endometrial thickness reached 8-14 mm in transvaginal sonography. Next, 100mg/day progesterone (progesterone ampule, 50 mg, Iran Hormone Co., Iran) were injected for 3-5 days based on the embryo stage. Estradiol valerate was continued until 3-5 days before the ET (depending on the stage of embryo development).

On the day of the transfer, class-A frozen embryos, after thawing, were re-evaluated for quality control, and those whose quality had declined below grade D were excluded from the research. The number of embryos that were transferred was based on the mother's age as the protocol (28).

The fetus was transferred to the uterus using a cook catheter (Cooper Surgical Co., USA)

For women younger than 35 yr and those aged 35-40 yr, two and three embryos with grade-A were transferred, respectively.

Then, participants were randomized into two groups, (n = 120/ each) as follows:

Group I received vaginal progesterone 400-mg/ twice a day (Cyclogest, vaginal progesterone (400 mg), Actavis, UK).

Group II received GnRH-agonist (0.1 mg triptorelin or dipherelin; Decapeptyl; IPSN, France) subcutaneously on the day of embryo transfer, and then three and six days after that + vaginal progesterone 400-mg/twice a day.

Our primary outcome included clinical pregnancy rates (CPRs), while the secondary outcomes included the overall conception rate, ongoing pregnancy rate (pregnancy beyond 12 wk), and the rate of spontaneous abortion in each group.

On the 16 ${}^{\mathrm{th}}$ day after the ET, the chemical pregnancy rate was investigated by a beta human chorionic gonadotropin (β-HCG) blood test. The clinical pregnancy rate was measured by a transvaginal sonography six wk after a positive β-HCG test for observing the fetal heart rate and a live intrauterine pregnancy.

Then, in the 12 ${}^{\mathrm{th}}$ wk of pregnancy, a transvaginal sonography was done again to confirm a continuation of live pregnancy (ongoing pregnancy). LPS was continued according to the protocol up to 12 wk after the ET. Also, participants were observed for spontaneous abortion during the first 20 wk of the pregnancy.

### Ethical considerations

The study protocol was approved by the Ethics Committee of Shiraz University of Medical Sciences (Code: IR.SUMS.MED.REC.1395.49) and registered at the Iranian Registry of Clinical Trials. Based on the available evidence from literature, we know that these two types of treatments do not pose any potential risk to participants. Informed consent was obtained from all participants prior to the initiation of treatment and all treatment procedures were explained to them.

### Statistical analysis

Statistical analysis was performed using SPSS software (Statistical Package for the Social Sciences, Chicago, IL) version 18. Data were presented as number (%) or mean ± SD/median as appropriate. Elementary and continuous variables were analyzed using Chi-square and Mann-Whitney tests. The relative risk (95% confidence interval) was calculated. P-value < 0.05 was considered as statistically significant.

## 3. Results

**Figure 1:**
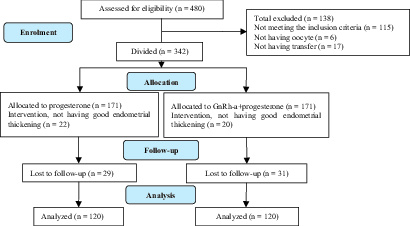
The study Consort diagram.

Of the 480 total cases with infertility, 115 failed to meet the inclusion criteria, 6 had no oocyte for retrieval, and 17 did not have any transfers during the research time. The remaining 342 cases were randomly divided into two groups: group I (n = 171): the vaginal progesterone suppository group and group II (n = 171): vaginal progesterone + GnRH-a group. Moreover, 22 cases in the group I and 20 in the group II did not have a good endometrial thickening and 29 cases in group I and 31 in the other group were lost to follow-up and were therefore excluded from the study. Finally, the data of 240 women (n = 120 in each group) were analyzed (Figure 1).

The two groups were matched in the terms of age, BMI, duration of infertility, infertility type, number of embryo transferred, embryo quality, endometrial thickness at the time of transfer, and infertility cause (Table I).

**Figure 2:**
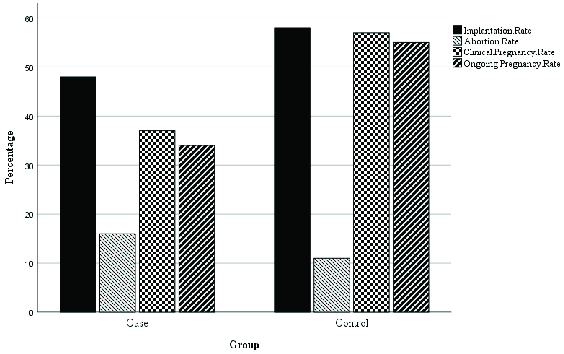
The implantation, clinical pregnancy, ongoing pregnancy, and abortion rates in patients.

Our findings showed no significant difference between the two groups in the terms of the implantation and abortion rates. But, the differences were significant in terms of clinical pregnancy and ongoing pregnancy rates (Table II, Figure 2).

No side effects have been reported with any medication in the two groups.

**Table 1:** Comparison of baseline characteristics in two study groups


		**Group I**	**Group II**	**p-value**
Age (yr)*		33.51 ± 5.09	33.28 ± 5.04	0.72 ${}^{a}$
Duration of infertility (yr)*		7.01 ± 4.57	7.31 ± 4.91	0.518 ${}^{b}$
Infertility cause**				
	**Tubal factor**	14 (11.7)	22 (18.3)	
	**Male factor**	3 (2.5)	1 (0.8)	
	**Unexplained**	35 (29.2)	36 (30)	
	**Premature ovarian failure**	18 (15)	18 (15)	
	**Polycystic ovarian syndrome**	50 (41.7)	43 (35.8)	0.506 ${}^{c}$
Type of infertility**				
	**Primary**	58 (48.2)	55 (45.8)		
	**Secondary**	62 (51.6)	65 (54.1)		0.698 ${}^{d}$
Body mass index (kg/m²)*		24.9 ± 2.2	23.8 ± 2.41			0.37 ${}^{a}$
Number of embryo transferred in the current cycle*		2.38 ± 0.61	2.45 ± 0.63	0.30 ${}^{b}$
Embryo quality (good quality - A)**		70 (58.3)	60 (50)	0.19 ${}^{d}$
Endometrial thickness at the time of transfer (mm)*		8.59 ± 0.79	8.54 ± 0.66	0.745 ${}^{b}$
*Data presented as Mean ± SD. **Data presented as n (%). ${}^{a}$ *t* test, ${}^{b}$ Mann-Whitney, ${}^{c}$ Fisher's exact-test, ${}^{d}$ Chi-square				

**Table 2:** Comparison of pregnancy outcomes in two study groups


	**Group I**	**Group II**	p-value*
**Implantation rate**	48 (40)	58 (48.3)	0.194
**Abortion rate**	16 (13.3)	11 (9.2)	0.307
**Clinical pregnancy rate **	37 (30.8)	57 (47.5)	0.008
**Ongoing pregnancy rate**	34 (28.3)	55 (45.8)	0.005
Data presented as n (%), *Chi-Square Test			

## 4. Discussion

The aim of this clinical trial study was to compare the effect of vaginal progesterone and multiple doses of GnRH-a + vaginal progesterone for LPS after an FET in the IVF cycles. Our results showed that GnRH-a + vaginal progesterone increases the pregnancy rate. However, no significant difference was noted in terms of implantation and abortion rates.

An LPS plays an important role in improving the IVF outcome. Nowadays, the use of exogenous progesterone is an inseparable step for supporting the luteal phase as it is based on multiple evidence of increased pregnancy and live births (24-26). However, it seems that the progesterone alone is not enough for LPS specially in an FET or IVF cycles, so GnRH-a was suggested as a adjuvant therapy in combination with progesterone for LPS (11-13).

Several studies have demonstrated that using GnRH-a in the luteal phase can increase the CPR, ongoing pregnancy rate, and live birth rate in assisted reproductive technology cycles. A series of studies have shown that an additional single dose of GnRH-a in the luteal phase can improve the result of IVF-ET in assisted reproductive technology cycles but only a few studies have compared the single vs. multiple doses of GnRH-a for LPS in FET cycles (29-34).

In the Yildiz and co-workers study, the effects of adding GnRH-a to the routine LPS was investigated and showed that the implantation, clinical, and ongoing pregnancy rates, and multiple pregnancies were increased. Also, two doses of GnRH-a (1 mg luperamide) on the 3 ${}^{\mathrm{rd}}$ and 6 ${}^{\mathrm{th}}$ day after ET showed higher rate of multiple pregnancies and ongoing pregnancy rate than a single injection on the 3 ${}^{\mathrm{rd}}$ day of ET (35). In Zafardoust's study, the effects of GnRH-a in the LPS has been investigated. Its administration with 0.1 mg of decapeptide six days after ET significantly increased the rate of implantation and pregnancy (36). Likewise, Oliveira's study showed that the administration of GnRH-a in the luteal phase led to an increase in the implantation rate (23). Kurng and colleague in their study concluded that the administration GnRH-a in LP improved pregnancy outcomes especially in cases with FSH > 8 miu/ml and in those with mature oocytes < 3/cycles (37).

Barltava and co-workers also reported that the continuation of daily GnRH-a for 2 wk in a fresh cycle and even in cases with first IVF failure can lead to satisfactory pregnancies (38, 39). Tesarik and colleagues reported a significant increase in the implantation rates with GnRH-a which was added to the routine LPS, which was the same result as our study and emphasized the useful effect of GnRH-a on embryo implantation. The observed effects of GnRH-a on the implantation and embryonic β-HCG secretion were attributed to a direct effect on the embryo or its effect on the endometrium through LH (40).

However, a few conflicting results on the beneficial effects of GnRH-a for LPS have also been reported (41-43). Ata and colleagues did not observe any beneficial effects of the use of GnRH-a as an LPS in patients stimulated by long-term luteal GnRH-a protocol (41). Davar and colleagues in their RCT administered 0.1 mg decapeptyle subcutaneously three days after the ET plus daily vaginal progesterone against the use of vaginal progesterone alone. They found no differences in the implantation, clinical pregnancy, ongoing pregnancy, and miscarriage rates (44).

Fortunately, no long-term side effects have been reported with GnRH-a in conception products or neonates (37, 45, 46). Li and others in their meta-analysis examined double doses of the GnRH-a in the LP of FET cycles, which was associated with increased fertility outcomes (47). In their meta-analysis, Song and co-workers showed the administration of a single dose of GnRH-a (5 to 6 days after IVF/ICSI procedures) for LPS significantly increased clinical pregnancy, ongoing pregnancy, and live birth rates compared to the control group (15). In our previous study, which evaluated three doses of GnRH-a as an LPS in FET cycles, the CPR was the highest in the group using the multiple doses of GnRH-a (0, 3, 6 days) (48).

The results of the current study showed that multiple doses of GnRH-a + vaginal progesterone as an LPS in FET cycles increases the pregnancy rate. In terms of CPR, a significant difference was noted between the two groups (p = 0.008). Also, for ongoing pregnancy rate (three months after an FET), a significant difference was observed between two groups (p = 0.005).

Although the mechanisms of action are not yet fully understood, GnRH-a does not interfere with the luteal phase and has a stimulating effect on the corpus luteum at certain doses (20).

The multiple doses of GnRH-a lead to gradual production of progesterone and E2 from the corpus luteum as a natural cycle. This effect is in contrast to the HCG that lead to a rapid increase in progesterone level and a premature decline in the implantation window (47). GnRH-a leads to adequate levels of luteal progesterone by acting on the pituitary gland to stimulate LH surge and preserve the corpus luteal (49). These multiple doses of the GnRH-a drug act as a bridge between the triggering period and the onset of HCG secretion by the implanting blastocyst (50). Luteal phase progesterone curve in multiple doses of GnRH-a cycles is similar to natural cycles. Therefore, the rapid endometrial growth that can lead to endometrial-embryonal dysregulation is eliminated in these cycles and the implantation rate could increase as a result. The clear effects of the GnRH-a on human morula have already been described.

In addition, in a recent Cochrane's meta-analysis, the live birth and ongoing pregnancy rates in the progesterone + GnRH-a group were higher than the progesterone-only group. There was no difference in abortion and multiple pregnancies in both groups (nine RCTs, 2,861 women) (18).

##  Limitations

The limitations of this study were the lack of alignment of patients, lack of the initial evaluation of patient's biochemistry for LH, FSH, and progesterone levels during the intervention, lack of follow-up until the birth time in the pregnant fertilized women, and not evaluating the side effects of the vaginal progesterone and diphereline. Further researches can reduce these limitations.

Finally, our findings showed that after the frozen embryos were transferred, taking 3 doses of 0.1-mg dipherline + daily vaginal progesterone instead of a daily use of 800-mg vaginal progesterone alone was associated with increased clinical and ongoing pregnancy rate, however, it did not show a significant difference in spontaneous abortion and implantation rate.

## 5. Conclusion

Using GnRH-a + vaginal progesterone as opposed to vaginal progesterone alone for LPS after IVF may be the superior choice and lead to higher rate of CPR and ongoing pregnancy rate.

##  Conflict of Interest

The authors declare that there is no conflict of interest.
